# Regulation of *P _suc_* , a sucrose-inducible promoter in *Caulobacter crescentus*

**DOI:** 10.17912/micropub.biology.001458

**Published:** 2025-02-25

**Authors:** Erin NewRingeisen, Jacy Jordahl, Lisa Bowers

**Affiliations:** 1 St. Olaf College, Northfield, Minnesota, United States

## Abstract

In gram-negative bacteria, when nutrients are too large or too scarce to diffuse through outer membrane porins, TonB-dependent receptors (TBDRs) are utilized to actively translocate substrates across the outer membrane. *Caulobacter crescentus* is a gram-negative bacterium with a large set of TBDRs, many which have not been fully characterized. Previous studies identified SucA, a *Caulobacter* TBDR that transports sucrose. Our experiments further characterize the expression of *sucA* from the *P _suc_* promoter and we identify *P _suc_* as a tightly controlled, tunable promoter, responsive to changes in sucrose concentration with and without other carbon sources in the media.

**Figure 1. Genomic neighborhood and expression of sucA in Caulobacter crescentus .**
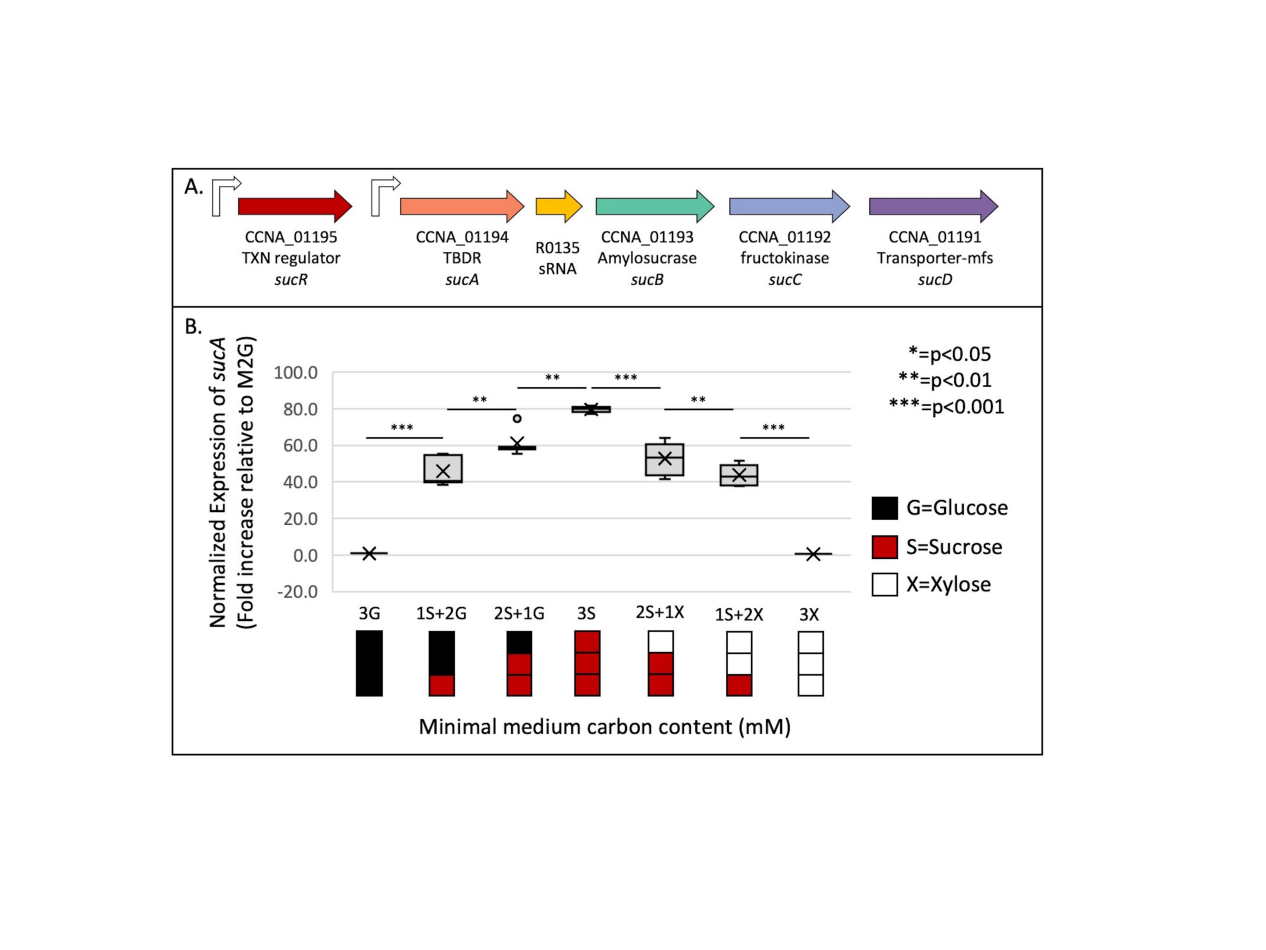
Figure 1. (A) Schematic of the *suc* locus in *Caulobacter crescentus* . The locus includes *P _suc_* followed by *sucABCD* . *P _suc_* is regulated by the transcriptional repressor SucR, encoded upstream from a constitutive promoter. (B) Expression of *sucA* from the *P _suc_* promoter in cells cultured with M2 minimal medium containing various concentrations of sucrose, glucose, and xylose, relative to cells grown in M2G. Abbreviations: (3G)=3.0 mM glucose; (1S+2G)=1.0 mM sucrose + 2.0 mM glucose; (2S+1G)=2.0 mM sucrose + 1.0 mM glucose; (3S)=3.0 mM sucrose; (2S+1X)=2.0 mM sucrose + 1.0 mM xylose; (1S+2X)=1.0 mM sucrose + 2.0 mM xylose; and (3X)=3.0 mM xylose. Real-time quantitative PCR (qRT-PCR) was performed on RNA extracted from the wild-type strain (NA1000). Calculation of relative expression includes normalization against the endogenous reference gene, 16s RNA. This assay was performed in three independent trials (each in technical triplicate). (*p-value 0.05, **p-value 0.01, ***p-value 0.001)

## Description

*Caulobacter crescentus* is a free-living, oligotrophic, gram-negative bacterium commonly found in soil and aquatic environments. Like most free-living oligotrophic bacteria, it likely encounters a variety of nutrients that are scarce or fleeting in its natural environment (Hentchel et al., 2019) . To respond to rapidly changing nutrient availability, cells tightly regulate a number of different mechanisms for nutrient uptake and metabolism.

*P _suc_* is a promoter that drives expression of the *suc* operon, a polysaccharide utilization locus including four genes ( *sucABCD* ) that are involved in the uptake and catabolism of sucrose in *Caulobacter crescentus* ( Figure 1A ). Previous studies determined that *P _suc_* is repressed by the constitutively expressed LacI-type repressor, SucR, and significantly induced in M2S media (where sucrose is the sole carbon source) compared to M2G media (where glucose is the sole carbon source) (Modrak et al., 2018) .

In contrast to typical laboratory-formulated minimal media, replete with a single carbon source, natural aquatic environments are complex and dynamic environments, often mixtures of dissolved sugars, including glucose, xylose, and sucrose (Rogers, 1965) in the µM range (Sogin et al., 2022) . Considering the natural complexity of aquatic environments, we investigated whether expression from *P _suc_* can be fine-tuned by the concentration of sucrose in the media and whether combinations of different carbohydrates affect expression from *P _suc_ .* We cultured WT *Caulobacter* in M2 minimal media salts, supplemented with mixtures of sucrose+glucose, such that the total carbon content for each media formulation was equivalent (3.0 mM). When the mixed-sugar cell cultures were in mid-log phase, we harvested the total RNA and used qRT-PCR to quantify expression of *sucA* (the first gene in the operon). As the concentration of sucrose in the mixed-media increased from 0.0, to 1.0 mM, 2.0 mM and 3.0 mM, the relative expression of *sucA* from the *P _suc_* promoter also increased in a step-wise fashion ( Figure 1B ). In these cultures, the proportion of sucrose and glucose were inversely related, meaning that as sucrose concentration increased, glucose concentration decreased. Therefore, in order to rule out any potential negative effect of glucose on expression from the *P _suc_* promoter in the mixed media, we repeated the expression experiments replacing glucose with xylose, another carbohydrate found naturally in aquatic environments. In the mixed sucrose+xylose cultures, we again observed a direct positive relationship between sucrose concentration and *sucA* expression, with expression from the *P _suc_* promoter at similar levels with both glucose and xylose as the second sugar ( Figure 1B ). These results support previous observations that *Caulobacter crescentus* does not exhibit significant effects of carbon catabolite repression in the presence of glucose (Meisenzahl et al., 1997) .

Thus, we determined that *P _suc_* is a tunable promoter, highly responsive to changes in sucrose concentration, and that expression from *P _suc_* is controlled positively by sucrose rather than negatively by glucose. This level of regulation would be advantageous for *Caulobacter* in environments with limited and fluctuating nutrient availability. These results also support the use of *P _suc_* as an inducible promoter for genes of interest in *Caulobacter* , along with the xylose-inducible *P _xyl_* and vanillate-inducible *P _van_* promoters (Meisenzahl et al., 1997; Thanbichler et al., 2007) . Sucrose is an inexpensive, stable, and readily-available inducer and because *P _suc_* is tightly regulated and can be tuned with various concentrations of sucrose with or without other carbon sources, it can be useful independently or simultaneously with *P _xyl_* or *P _van_* to allow controlled overproduction or depletion of genes of interest.

## Methods

**Gene expression assay:** *Caulobacter crescentus* NA1000 cells were incubated in M2 minimal medium (Ely, 1991) , supplemented with 3.0 mM D-glucose (M2G) overnight at 30°C with aeration, then cells were washed twice and sub-cultured at a starting OD _660_ =0.05 into 50 mL M2 minimal medium supplemented with the following sugars:

**Table:** 

3G	1S+2G	2S+1G	3S	1S+2X	2S+1X	3X
3.0 mM glucose	1.0 mM sucrose 2.0 mM glucose	2.0 mM sucrose 1.0 mM glucose	3.0 mM sucrose	1.0 mM sucrose 2.0 mM xylose	2.0 mM sucrose 1.0 mM xylose	3.0 mM xylose

Subcultures were incubated for 4 hours with aeration and RNA was harvested for qRT-PCR using the PureLink RNA minikit (Invitrogen) and treated with RNase-free DNase I (Promega). The DNase was then removed with a second round of the PureLink RNA minikit. qRT-PCR amplification was performed with a Rotor-Gene Q thermocycler (Qiagen). Reactions were performed in 20μl volumes with 100ng total RNA per sample. GoTaq 1-step qRT-PCR system (Promega) was used to reverse transcribe the RNA and amplify the target sequence using target specific primers. The 16S rRNA gene was used as the reference gene. All primer pairs were validated for amplification efficiency (absolute value of the slope of ΔCt vs LOG ng template is < 0.1) and relative quantification of gene expression analysis was carried out with the comparative Ct method (ΔΔCt). In this method, the ΔCt values were calculated by ΔCt= Ct(target gene) – Ct(reference gene), and the ΔΔCt values were calculated by ΔΔCt=ΔCt(test sample) – ΔCt(calibrator sample). Fold change of the target gene was calculated by 2^ ^(-ΔΔCt)^ .qRT-PCR experiments were carried out in at least three independent biological assays, each with three technical replications.

## References

[R1] Ely Bert (1991). [17] Genetics of Caulobacter crescentus. Methods in Enzymology.

[R2] Hentchel Kristy L, Reyes Ruiz Leila M, Curtis Patrick D, Fiebig Aretha, Coleman Maureen L, Crosson Sean (2018). Genome-scale fitness profile of
*Caulobacter crescentus*
grown in natural freshwater. The ISME Journal.

[R3] Meisenzahl A C, Shapiro L, Jenal U (1997). Isolation and characterization of a xylose-dependent promoter from Caulobacter crescentus. Journal of Bacteriology.

[R4] Modrak Samantha K., Melin Martha E., Bowers Lisa M. (2018). SucA-dependent uptake of sucrose across the outer membrane of Caulobacter crescentus. Journal of Microbiology.

[R5] Rogers M.A (1965). Carbohydrates in aquatic plants and associated sediments from two Minnesota lakes. Geochimica et Cosmochimica Acta.

[R6] Sogin E. Maggie, Michellod Dolma, Gruber-Vodicka Harald R., Bourceau Patric, Geier Benedikt, Meier Dimitri V., Seidel Michael, Ahmerkamp Soeren, Schorn Sina, D’Angelo Grace, Procaccini Gabriele, Dubilier Nicole, Liebeke Manuel (2022). Sugars dominate the seagrass rhizosphere. Nature Ecology & Evolution.

[R7] Thanbichler M., Iniesta A. A., Shapiro L. (2007). A comprehensive set of plasmids for vanillate- and xylose-inducible gene expression in Caulobacter crescentus. Nucleic Acids Research.

